# Circulating Tumor Cells: Pathological, Molecular and Functional Characteristics

**DOI:** 10.3390/ijms25158198

**Published:** 2024-07-27

**Authors:** Ewa A. Grzybowska

**Affiliations:** Maria Sklodowska-Curie National Research Institute of Oncology, Roentgena 5, 02-781 Warsaw, Poland; ewa.grzybowska@nio.gov.pl

## 1. Introduction

This Special Issue, ‘Circulating Tumor Cells: Pathological, Molecular and Functional Characteristics 1.0’, addresses several issues concerning the diagnostic power and molecular characteristics of circulating tumor cells (CTCs). This field is expanding rapidly, and some of its latest advances, concepts, and perspectives are described below.

### 1.1. The Importance of CTC Enumeration and Molecular Analysis in the Clinic and for Basic Research

The enumeration and molecular analysis of circulating tumor cells (CTCs) is gaining importance in both clinical studies and in basic research concerning the mechanisms of metastasis. In clinical settings, CTCs have been proposed as new blood biomarkers, potentially being more reliable and prognostic than classic biomarkers used for diagnosis, tumor stage assessment, therapy choice, response to treatment, and prognosis (e.g., carcinoembryonic antigen [CEA], squamous cell carcinoma antigen [SCCA], prostate specific antigen [PSA], lactate dyhydrogenase [LDH], cancer antigens: CA125, CA15-3, CA19-9, and others) [1]. The CTC count has been shown to be prognostic and predictive in many types of cancer [2,3,4,5,6,7,8,9]; however, most of the research has been carried out on breast cancer. The utility of CTC monitoring is tested in the early postoperative stages, in which it can be interpreted as a warning sign for progression to metastatic disease [10,11,12]; in a metastatic setting, CTC monitoring can be used as a tool for disease monitoring and a possible signal for treatment changes [11,13]. Large clinical trials have been designed to include the CTC count in clinical use, and their results have already demonstrated the efficacy of this approach [14].

However, the enumeration of CTCs utilized in clinical studies represents only one aspect of CTC studies. The molecular analysis of CTCs is currently not included in clinical practice, but it contributes to our knowledge of metastatic disease, and may advance to a clinical setting. CTCs represent the active phase of metastasis and a small proportion of them (0.01%, ref. [15]) lead to the establishment of secondary lesions. However, even if they are not destined to seed metastases, their genetic makeup and phenotypic plasticity may provide us with important information about the course of the disease. An insight into the biology, genotype, epigenetic changes, phenotype, and the expression profile of isolated CTCs offers a great chance to explore tumor evolution in real time, with the continuous monitoring of the factors contributing to both the resistance and vulnerabilities of the metastatic lesions. It has been observed that metastatic disease can vary considerably from primary tumors [16]; molecular studies on CTCs can provide information on the current state of tumor cells in a specific patient, which could be a big step towards their use in personalized medicine.

It is worth noting that CTCs are extremely heterogeneous, genetically, epigenetically, and phenotypically. This prompts the question whether the heterogeneity results from the similar heterogeneity of the primary tumor, or whether it evolved during tumor progression. A further analysis of the differences in CTCs versus the matching primary tumor samples should clarify this issue.

Another related key question is if it is possible to pinpoint specific molecular features of CTCs that predestine them to give rise to metastases so as to target this subpopulation therapeutically. The common assumption is that these “seeders” should have an increased expression of stem cell and mesenchymal markers. However, there are indications that it is not the fully mesenchymal characteristics, but rather the hybrid epithelial–mesenchymal characteristics (partial EMT phenotype) that are more metastatic [17,18,19].

The majority of CTCs is represented by single cells, but they also occur as clusters, both homotypic (composed of the same tumor cells) or heterotypic (composed of tumor cells and other cell types, mostly immune cells). Clusters have been shown to have a much higher metastatic potential [20,21,22] to the point of denying the ability of single CTCs to seed metastasis [23].

Further research should comprehensively resolve the role of CTCs in the metastatic cascade, which is bound to serve in the combat against metastatic disease.

### 1.2. Different Approaches to CTC Detection and Isolation

CTC analysis is more convenient and comfortable for patients than secondary tumor biopsy or disseminated cell analysis, but it presents challenges of its own. Currently, there are two FDA-approved systems for CTC detection: CellSearch^TM^, an immunolabeling-based method, and Parsortix^TM^, a size-based method. CellSearch^TM^ dominates the field, but its well-known weakness is its dependence on EpCAM expression; this is an issue because not every tumor expresses EpCAM, and even if it does, there is considerable heterogeneity in EpCAM expression between CTCs, even from the same patient. 

Apart from these two main systems, many other methods are employed, differing in principle, efficacy, and specificity, but they can be used only for research.

Differences in the methodology for the identification of CTCs can lead to conflicting reports that describe their status. Thus, it is crucially important to establish the best, standardized, and validated methods for CTC identification and analysis. The challenge is not only to choose the best, but also to tailor a specific method to a specific task. Clinical purposes may require a different approach than experimental ones, and different types of cancer, divergent in marker expression and phenotypic features, may require different methods of CTC identification.

Moreover, the molecular analysis of isolated CTCs presents other difficulties. CTCs represent extremely difficult material, as they are rare, require reliable identification and isolation, and contain only picograms of DNA or RNA. Therefore, molecular analysis requires an amplification of the material, which may introduce bias and artifacts. Additionally, some methods, especially size-based ones, have been shown to damage some of the isolated cells [24].

These notions demonstrate how challenging the molecular analysis of CTCs is and should therefore be considered when comparing results obtained using different methods.

## 2. Heterogeneity and Biomarker Conversion in Breast Cancer

Breast cancer is relatively well studied with regard to the number of known CTCs, characteristics, and the clinical implications of these data compared to other types of cancer. To add to this information, Kaur et al. [25] describe the feasibility of exome sequencing in CTCs versus metastatic biopsies in BCs. The whole genome sequencing approach allowed the authors to determine the level of genomic concordance for somatic nucleotide variants, copy number alterations, structural variants, and chromosomal rearrangements between CTC and metastases, adding to the repertoire of possible molecular approaches. The authors observed a high level of somatic alterations and molecular heterogeneity in their samples, including the most prevalent focal deletion events. However, the study’s main input was the intension to show the feasibility of such a wide-range approach in the context of such rare and difficult material.

Most types of breast cancer are hormone-dependent and exhibit a high expression of estrogen receptors (ER) (luminal subtypes). Estrogens promote the proliferation of breast cancer cells, and thus anti-estrogen therapies are highly effective. However, the ER status can change during therapy, conferring resistance to treatment, which can lead to an increased proliferation of the remaining tumor cells which are no longer blocked by anti-estrogens, and subsequently, to metastasis. Taking into account the high heterogeneity of primary tumors, it is still unclear at what stage of cancer progression endocrine resistance occurs and how it evolves. Does it already exist in a small percentage of cells that constitute primary tumors and primes these cells for survival, or does it appear in later stages, in CTCs or disseminated tumor cells, possibly induced by endocrine therapy?

Forsare et al. [26] addressed this question by evaluating the status of the ER in primary tumor, CTCs, and disseminated cells at specific time points during the therapy. They observed a significant shift from ER positivity to negativity between the primary tumor (PT) and distant metastases (DM), but also CTCs. The presence of a CTC that showed positive for ERs was associated with a better prognosis. This indicates that the ER status evolves during progression, after tumor cells enter the bloodstream.

Stefanovic et al. [27] adopted another approach to biomarker conversion in metastatic breast cancer. The authors analyzed the number of CTCs in subgroups with and without changes in biomarker expression and did not find significant differences, except for the observation that CTCs were detected less frequently in patients with stable HER2 expression. 

The heterogeneity of breast cancer cells was also observed by Savelieva et al. [28], but from the angle of stem cells and EMT markers in detected CTCs. The assumption was that CTCs that display stem cell markers and mesenchymal characteristics are more likely to become metastatic “seeds”. The analysis revealed that the heterogeneity of stem marker expression in CTCs was observed regardless of N-cadherin expression, although all N-cadherin+ CTCs demonstrated stem cell characteristics, and N-cadherin expression was found to be associated with the expression of the stromal cell-derived factor-1 (SDF-1) receptor. 

## 3. Alternative Technical Approaches and Markers in CTC Research

Breast cancer belongs to the group of tumors expressing EpCAM; therefore, it can be analyzed using EpCAM-dependent systems, mainly via CellSearch^TM^. However, for some tumors, other approaches are necessary, as these tumors do not express specific markers commonly used for CTC detection/isolation. Renal cancer falls into this category. Cappelletti et al. [29] provided an analysis in which they overcame this limitation using the marker-independent Parsortix™ technique coupled with DEPArray™, utilizing single cell isolation and a subsequent molecular analysis. With this approach, they characterized the two CTC subpopulations: epithelial CTC and non-conventional CTC (lacking epithelial and leukocyte markers). They also identified metastasis-driving subclonal alteration in their samples.

EpCAM remains the main marker used for detection and isolation, but, as described above, it has limitations; cancer cells may not express EpCAM or its expression in CTCs may be lost due to epithelial–mesenchymal transition [30]. Sand et al. [31] describe an intriguing new assay for the clinical detection of CTC, based on the recombinant malaria protein VAR2CSA (rVAR2). This protein binds to a unique modification of chondroitin sulfate present in most cancer cells. Thus, the authors consider it to be a nearly universal tumor-cell-targeting reagent to isolate CTCs, enabling a novel EpCAM-independent approach.

## 4. Discussion Points

The metastatic potential of CTCs in breast cancer was addressed in several review articles of this Special Issue; the topics discussed included the difference between single CTCs and clustered CTCs, considering clusters that exhibit an increased metastatic capacity and better survival (Amintas et al. [32]), the heterogeneity of tumor cells as a factor contributing to metastasis (Menyailo et al. [33]), and CTC fusion with macrophages, which can lead to chemotherapeutic resistance and immune tolerance (Manjunath et al. [34]). The biology and prognostic value of CTC in early and advanced BC was summarized by Fabisiewicz et al. [35].

## 5. Conclusions

CTC research is continually progressing, and its results are already being used in clinical practice; however, we still need to refine its parameters and techniques, complete clinical trials, and fine-tune therapeutic responses. Molecular studies on isolated CTCs should enable us to use this information in precision diagnostics and personalized treatments of the future, as well as to gain a general knowledge of the mechanisms of metastasis. 
